# In This Issue

**Published:** 1999

## WHAT IS CRAVING? MODELS AND IMPLICATIONS FOR TREATMENT

Many abstinent alcoholics experience craving for alcohol, sometimes long after becoming sober. Researchers have developed several models to determine what prompts this insidious urge for alcohol. Dr. Raymond F. Anton reviews those models, including a neuroadaptive model which suggests that long-term exposure to alcohol induces changes in the brain that lead to craving. Investigators have proposed numerous brain regions that may be involved in this neuroadaptation and which thus may contribute to craving. These regions include the brain’s reward center (i.e., the nucleus accumbens) and regions of the frontal cortex that integrate incoming sensory information. Despite the progress that has been made in understanding the basis of craving, however, additional research is needed to develop more accurate ways of measuring craving’s intensity. (pp. 165–173)

## INDUCING CRAVING

Why do people drink? What are the best methods for treating and preventing alcoholism? Investigators believe that the solutions to those questions may be found, in part, in the mechanisms underlying craving. To better understand those mechanisms, researchers have developed techniques to induce craving for alcohol in a controlled laboratory setting. Dr. Mark D. Litt and Mr. Ned L. Cooney describe methods for inducing craving, such as presenting subjects with actual alcoholic beverages, exposing them to cues that stimulate drinking (e.g., alcoholic beverage advertisements), or inducing certain mood states (e.g., anxiety or anger). Craving’s intensity can be rated subjectively by the patient or assessed clinically by measuring the patient’s behavior or physiological responses. Attempts to induce craving in the laboratory have been only moderately successful. The authors conclude their article by suggesting more effective ways of inducing craving. (pp. 174–178)

## ASSESSING CRAVING FOR ALCOHOL

Accurately assessing a person’s mental state is inherently difficult, primarily because it relies on that person’s subjective report of how he or she feels. Ideally, self-report instruments used for craving assessment should explore key aspects of the craving phenomenon; be readily measurable (that is, have good psychometric characteristics, such as reliability and validity); and take into account a specific timeframe. Several such questionnaires are currently available, report Drs. David J. Drobes and Suzanne E. Thomas. In addition, researchers and, to a lesser extent, clinicians are using behavioral measures (e.g., amount of alcohol consumption) and psychophysiological variables (e.g., changes in salivation, skin temperature, and heart rate) to assess the full range of responses that may be related to craving. The increased use of such approaches may help improve alcoholism treatment. Although investigators are only beginning to explore the potential of these new technologies for visualizing craving for alcohol and other drugs, brain regions already have been identified that show a response to craving-inducing stimuli. (pp. 179–186)

## FUNCTIONAL IMAGING OF CRAVING

Craving is primarily an emotional experience or mental state that can be conveyed to other people only through self-reports. Unfortunately self-reports vary considerably from patient to patient, making research of this phenomenon highly complicated. One method that may help researchers in measuring an experience such as craving is the development of functional imaging techniques. These images provide snapshots of the changes that occur in brain activity in response to craving-inducing stimuli. Dr. Daniel W. Hommer reviews several promising imaging techniques, including single photon emission computed tomography, positron emission tomography, and functional magnetic resonance imaging. (pp. 187–196)

## APPROACHING AVOIDANCE

The concept of craving is useful in understanding and treating alcoholism. However, craving is but one component of the array of mental processes that influence drinking behavior. Drs. Mary Jo Breiner, Werner G. K. Stritzke, and Alan R. Lang suggest that the decision to take a drink is the result of a competitive balance between inclinations to approach drinking and inclinations to avoid drinking. In this view, alcoholics are seen as both drawn toward and repelled from alcohol use. Understanding the dynamics of a patient’s ambivalence toward alcohol consumption may help clinicians motivate the patient to stay abstinent by influencing the importance that the patient assigns to the positive and negative outcomes of alcohol consumption. (pp. 197–206)

## MEDICATIONS AND ALCOHOL CRAVING

High levels of craving are sometimes associated with relapse following alcoholism treatment. Research suggests that medications which reduce craving may improve alcoholism treatment. Four medications are currently marketed for treating alcoholism: disulfiram (Antabuse^®^), naltrexone (ReVia^™^), acamprosate, and tiapride. Dr. Robert M. Swift evaluates the effects of these and other medications on drinking behavior and on craving. A better understanding of the neurobiology of craving and the use of different medications in combination may further increase the effectiveness of the pharmacological treatment of alcoholism. (pp. 207–213)

## COGNITIVE CONCEPTS OF CRAVING

In recent years, researchers have turned to cognitive concepts (such as learning, information processing, problem-solving, and decisionmaking) and cognitive methodology (such as dual-task procedures in which a subject is asked to perform two tasks simultaneously) in an attempt to better understand craving. Dr. Stephen T. Tiffany reviews a model of the cognitive processes underlying craving. This model postulates that although many of the behaviors involved in seeking and using alcohol have become automatic in experienced alcoholics, craving is a nonautomatic process that requires concentrated effort. This theory has significant implications. Because a person’s cognitive resources are limited, an alcoholic faced with craving will be less able to cope with other day-to-day cognitively demanding situations. This would account for the disruptive impact that craving has been observed to have on the everyday functioning of some alcoholics. (pp. 215–224)

## DOES URGE TO DRINK PREDICT RELAPSE AFTER TREATMENT?

What role does craving play in causing a person to relapse to drinking? Drs. Damaris J. Rohsenow and Peter M. Monti review a number of models that have been developed to explore the link between craving and relapse. To measure the extent of craving, researchers and clinicians use methods such as role-play assessment and cue-reactivity assessment. Studies using these and other techniques have shown that urges can indeed predict subsequent relapse, particularly when a person is tested at the end of treatment using role plays of a simulated high-risk situation (such as being offered a drink). Other studies have found, however, that urges can act as warning signals for the drinker, alerting him or her of the need to increase coping skills in order to effectively deal with such risky situations. (pp. 225–232)

## ANIMAL MODELS OF CRAVING

Animal studies have advanced alcohol-craving research by providing information about the behaviors that are typically associated with craving. However, animal models of craving also have limitations. To help gain perspective on the use of animal models in craving research, *Alcohol Research & Health* invited a panel of leading researchers to comment on this topic. In a roundtable discussion format, Drs. George F. Koob, Friedbert Weiss, Stephen T. Tiffany, Walter Zieglgansberger, and Rainer Spanagel discuss the concept of craving, how animal models are used to test various theories of craving, and the benefits and limitations of using animal models to study human alcohol craving. (pp. 233–236)

